# Mortality Related to Cold Temperatures in Two Capitals of the Baltics: Tallinn and Riga

**DOI:** 10.3390/medicina55080429

**Published:** 2019-08-02

**Authors:** Daniel Oudin Åström, Triin Veber, Žanna Martinsone, Darja Kaļužnaja, Ene Indermitte, Anna Oudin, Hans Orru

**Affiliations:** 1 Division of Sustainable Health, Umeå University, 901 87 Umeå, Sweden; 2 Institute of Family Medicine and Public Health, University of Tartu, Ravila 19, 50411 Tartu, Estonia; 3 Physiotherapy and Environmental Health Department, Tartu Health Care College, Nooruse 5, 50411 Tartu, Estonia; 4 Institute of Occupational Safety and Environmental Health, Riga Stradinš University, Dzirciema 16, LV-1007 Riga, Latvia

**Keywords:** temperature-related mortality, distributed lag non-linear models, cold-related attributable fraction, winter mortality, Baltics, all-cause mortality and cause-specific mortality

## Abstract

*Background and objectives:* Despite global warming, the climate in Northern Europe is generally cold, and the large number of deaths due to non-optimal temperatures is likely due to cold temperatures. The aim of the current study is to investigate the association between cold temperatures and all-cause mortality, as well as cause-specific mortality, in Tallinn and Riga in North-Eastern Europe. *Materials and Methods:* We used daily information on deaths from state death registries and minimum temperatures from November to March over the period 1997–2015 in Tallinn and 2009–2015 in Riga. The relationship between the daily minimum temperature and mortality was investigated using the Poisson regression, combined with a distributed lag non-linear model considering lag times of up to 21 days. *Results:* We found significantly higher all-cause mortality owing to cold temperatures both in Tallinn (Relative Risk (RR) = 1.28, 95% Confidence Interval (CI) 1.01–1.62) and in Riga (RR = 1.41, 95% CI 1.11–1.79). In addition, significantly increased mortality due to cold temperatures was observed in the 75+ age group (RR = 1.64, 95% CI 1.17–2.31) and in cardiovascular mortality (RR = 1.83, 95% CI 1.31–2.55) in Tallinn and in the under 75 age group in Riga (RR = 1.58, 95% CI 1.12–2.22). In this study, we found no statistically significant relationship between mortality due to respiratory or external causes and cold days. The cold-related attributable fraction (AF) was 7.4% (95% CI -3.7–17.5) in Tallinn and 8.3% (95% CI -0.5–16.3) in Riga. This indicates that a relatively large proportion of deaths in cold periods can be related to cold in North-Eastern Europe, where winters are relatively harsh.

## 1. Introduction

With ongoing climate change, the health effects of heat waves and high temperatures have been thoroughly documented throughout the world [1], in Europe [2,3,4], and specifically in Estonia [5]. Even though it is likely that more frequent heat waves will occur in the future, the climate in the Baltics is generally cold, and the majority of deaths due to non-optimal temperatures in these areas are likely due to cold temperatures, as has been reported for neighboring countries like Sweden [6] and Finland [3]. The impact of cold temperatures on mortality is a phenomenon that is not exclusive to northern countries. It has also been reported across different temperature zones, where recent studies have shown that more temperature-attributable deaths are caused by cold than by heat [7,8,9], and effect estimates (relative risks—RRs) for cold are higher than those for heat [10,11].

The main adverse health effects that are associated with cold temperatures are cardiovascular and respiratory diseases [12,13]. Cold air has been observed to trigger vasoconstriction and bronchoconstriction, change endothelial function, and to increase blood viscosity, blood pressure and cardiac oxygen demand, leading to increased cardiovascular and respiratory hospitalization and mortality [12,14,15,16,17,18]. Estimated associations between cold days and mortality are stronger for older people [12,13,19]. Specifically in the region, Orru and Åström [20] revealed that deaths due to external causes (traffic accidents, assaults, fires) increase during extremely cold periods.

More detailed information about the effects of cold temperatures on mortality is essential for planning interventions to prevent cold-related deaths in the Baltic countries. As mentioned, the effect of temperature on mortality can vary substantially by geographical location because of the different climactic conditions, socioeconomic conditions and demographic characteristics of the population [7,10,11,12,13,21]. However, knowledge about the impact of cold temperatures on mortality in Estonia and Latvia is lacking.

Thus, the main aim of the current study is to investigate the association between cold temperatures and all-cause, as well as cause-specific, mortality in two capitals in the Baltics and to investigate if advanced age (75+) modifies the relationship. In addition, we will calculate mortality attributable to non-optimal temperatures during the cold season. 

## 2. Materials and Methods

**Figure 1:**
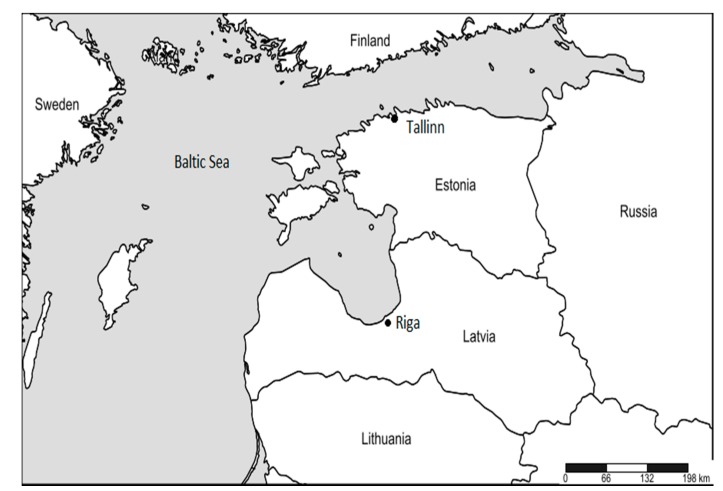
The study area and neighboring countries.

Estonia and Latvia are situated in North-Eastern Europe and are bordered by the Baltic Sea, Finland, the Russian Federation and Lithuania. These countries are in the northern part of the temperate climate zone and in a transition zone between maritime and continental climates. According to the Köppen-Geiger [22] classification of climatic zones, the area constitutes deciduous broadleaf forest (Dbf). In this study, we collected data from the capital of Estonia, Tallinn, and the capital of Latvia, Riga (Figure 1). 

Daily temperature data were acquired from the Estonian Weather Service (Tallinn, Estonia) and the Latvian Environment, Geology and Meteorology Centre (Riga, Latvia) for the five winter months (from November to March) for the periods of 1997–2015 (Tallinn, Estonia) and 2009–2015 (Riga, Latvia). We used the minimum daily temperature as the exposure variable. Temperature data were complete for both meteorological stations.

We collected daily all-cause mortality data for the same periods for the two cities respectively. Data on deaths were acquired from the Estonian Causes of Death Register and the Latvian Register of Causes of Death. The study period was selected based on the availability of digital data in registries. We used all-cause mortality in the main analysis, since cold temperatures have been shown to have significant impacts on external-cause mortality in the region, and excluding it may thus underestimate the total effect of cold temperatures on mortality [20]. Furthermore, we collected daily respiratory (ICD 10 J00–J99), cardiovascular (ICD 10 I00–I99) and external-cause (ICD 10 V00–Y99) mortality counts. 

We analyzed the short-term association between temperature and mortality using an overdispersed Poisson regression model. Poisson regression models with a stratum variable for date have been shown to yield identical results to those from a conditional logistic regression, which are used in a case-crossover study [23,24]. We additionally controlled for national public holidays. 

To allow for the effects of temperature on mortality that are delayed in time as well as non-linear, we used a distributed lag non-linear model (DLNM) [25]. We used lag structures of up to 21 days, allowing for delayed effects on mortality [7].

For temperature and time lag, we fit a quadratic B-spline and a natural cubic spline, respectively. For temperature, two equally spaced internal knots were used and two equally spaced knots were placed on the log-scale on the time scale. 

The cold season was defined as 1 November to 31 March. The results are presented for the cold season as a percentage increase in mortality using the minimum mortality temperature (MMT)—i.e., the temperature associated with the lowest rate of mortality—as a reference. To limit the influence of extreme temperatures on the MMT, we restricted the MMT to be found between the 5th and 95th percentiles of the seasonal (November to March) temperature distribution.

We calculated the total attributable fraction (AF) of mortality due to non-optimal temperatures for Tallinn and Riga and quantified the contribution of cold to mortality below the MMT. We also calculated the AF of mortality due to modestly and extremely cold days. Modestly cold days were considered to be temperatures between the MMT and the 2.5th percentile and extremely cold days were those below the 2.5th percentile [6,7].

We performed model checks by the visual inspection of normally distributed residuals. All analyses were performed with R version 3.4.0, and we used the DLNM package [26]. The study was conducted in accordance with the Declaration of Helsinki, and the initial protocol was approved by the Research Ethics Committee of the University of Tartu (197T-9), approved on 18th of October, 2010.

## 3. Results

As Tallinn and Riga are situated 280 km from each other on the coast of the Baltic Sea, the meteorological data show fairly similar temperatures between the two capitals (Table 1). In general, the minimum temperature was 5 degrees lower and the mean temperature 1.9 degrees lower in Tallinn as compared to Riga. During the study period, the coldest month was February (min/mean temperatures −27.3/−6.5 °C in Tallinn and −24.4/−4.9 °C in Riga), followed by January (−29.4/−5.9 °C and −23.1/−5.8 °C). The warmest months were November (−13.5/0.2 °C and −13.8/2.9 °C) and March (−21.2/−4.2 °C and −11.7/−1.0 °C). In December, the min/mean temperatures were −24.3/−3.4 °C in Tallinn and −15.3/−1.9 °C in Riga.

In total, there were 36,645 deaths in Tallinn and 27,495 deaths in Riga during the study period (Table 2).

The MMT was similar in Tallinn and Riga at 4.0 °C and 4.4 °C, respectively (Table 3). The MMT was found at the 94.2th and 89.6th percentiles of the cold season temperature distribution in Tallinn and Riga, respectively. We present the results as the cumulative relative risk (RR) over lags of 0 to 21 days, at the 5th percentile of the city-specific temperature distribution, using the city-specific MMT as a reference. 

**Figure 2:**
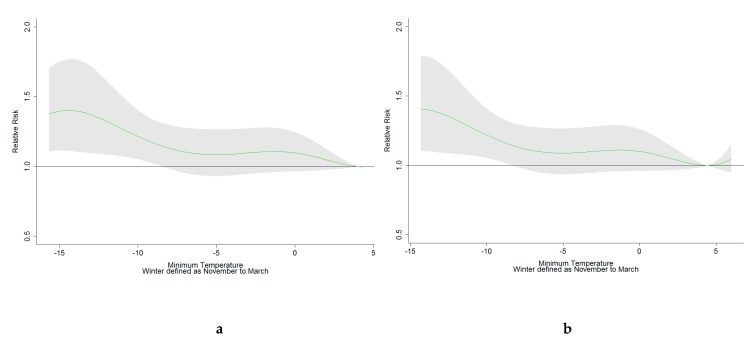
Cumulative effect over lags of between 0 and 21 days for the winter months (November to March) in Tallinn (**a**) and Riga (**b**) (shading 95% CI).

In our analyses, we could see that cold temperatures significantly increased all-cause mortality both in Tallinn and in Riga. In Tallinn, all-cause mortality increased by 28% (95% Confidence Intervals (CI): 1–62%), and, in Riga, mortality increased by 41% (95% CI: 11–79%) (Table 3). All-cause mortality increased slowly below the seasonal MMT in both cities. Due to the choice of the function used to describe mortality, the increase is not entirely linear with similar effects between 0 and −5 °C (Figure 2). In addition, significantly increased mortality due to cold temperatures was observed in the 75+ age group and among cardiovascular mortality in Tallinn and in the under 75 age group in Riga. Respiratory and external-cause mortality increased as well, but not to a significant extent.

Most of the cold mortality burden occurred during modestly cold days rather than extremely cold days. The total mortalities attributable to temperatures below the MMT were 7.4% (95% CI -3.7–17.5) in Tallinn and 8.3% (95% CI -0.5–16.3) in Riga. The AFs for modestly cold temperatures were 6.9% (95% CI -4.1–16.3) and 7.8% (95% CI -0.5–15.1) and, for extremely cold temperatures, they were 0.6% (95% CI -0.2–1.2) and 0.6% (95% CI -0.3–1.4), in Tallinn and Riga respectively.

## 4. Discussion

In our analysis, we could see a substantial effect of cold temperatures on mortality. Colder temperatures than the MMT during the cold season significantly increased all-cause mortality, both in Tallinn and in Riga. The all-cause mortality relative risks in Tallinn (RR = 1.28, 95% CI 1.01–1.62) are consistent with relative risk assessments in a neighboring country, Finland, where researchers identified all-cause mortality risks (RR = 1.26 and RR = 1.20) in two investigated districts [3]. In Riga, the all-cause mortality risk during the 21-day period (RR = 1.41, 95% CI 1.11–1.79) was higher than in Tallinn and in Finland, albeit not to a statistically significant extent.

Several modifiers to cold-related mortality have been suggested: macroeconomic factors (gross domestic product (GDP)); socioeconomic conditions (income poverty, income inequality, multiple deprivation, fuel poverty); household thermal efficiency; public health expenditure as a proportion of per capita GDP; education level; and influenza immunization status [19,27]. The observed differences in point estimates between Riga and Tallinn are probably not due to climate, because climate conditions in these neighboring countries are similar, and both cities are near the sea. However, the life expectancy, GDP and the share of current health expenditure in GDP during the study period were lower in Latvia than in Estonia [28]. The role of other risk factors (e.g., stress, alcohol use, access to health care and social guarantees) might be somewhat higher there, as large health inequalities still exist in Latvia [29], and the effect of cold might not be that prevalent.

Among cause-specific mortalities, this study confirms that cold temperature is associated with cardiovascular mortality, but this effect was demonstrated only in Tallinn and not to a significant extent in Riga. Cold stress promotes an increase in blood pressure and cardiac oxygen demand. Both of these causes may lead to an increase in the number of deaths due to cardiovascular reasons [12,15,16,18]. The finding of increased mortality during cold days due to cardiovascular diseases is shown in several studies (e.g., meta-analysis of cold spells by Ryti et al. [12]). Among cardiovascular diseases, acute myocardial infarctions and strokes are often related to cold temperatures [16,30]. In general, cardio-vascular mortality is very high in both cities, being somewhat higher in Riga than in Tallinn (3.3 and 2.5 cases per 1000 inhabitants during the winter period, respectively).

Contrary to previous studies, which have suggested an increase in mortality due to respiratory diseases during and after cold days [8,11,12,13,18], this study has been unable to demonstrate temperature associations with respiratory mortality in Tallinn and in Riga. In both cities, respiratory mortality increased during and after the cold, but we could not see any statistically significant effects. In the winter season, in this climate zone, a seasonal increase in respiratory tract infections prevalence has been described [28]. A possible explanation for this might be that, in general, the standardized mortality rates of respiratory diseases are much smaller in Estonia and Latvia compared to southern areas of Europe [31]. Similar results were found in Vilnius (Lithuania), where most cold-related mortality is due to circulatory rather than respiratory causes. Researchers explain that the population of Vilnius is well adapted to low temperatures, as opposed to residents of hot regions with lower physical, social and behavioral adaptation to low temperatures [32].

We did not find any statistically significant association between cold temperatures and external causes of death. Previously, Orru and Åström [20] reported that cold temperatures were associated with an increased risk of external-cause mortality. However, cold in that study was defined based on year-round data and not only on the winter months, as in the current study. In addition, a much longer lag structure was used in the current analysis, which may have impacted the results, since mortality due to external causes is not expected to have such a delayed effect. In general, in Latvia and Estonia, deaths due to external causes (traffic accidents, assault, fires) are the third largest cause of mortality after cardiovascular diseases and cancer, and thus any effect on external causes would have significant impacts on public health [28]. The effect of cold on external causes was also modified by an accident in Riga on 21 November, 2013, when the roof of a shopping mall collapsed, and 54 people died. It increased dispersion in the group of external causes and especially in the 0–74 age group.

The results in Tallinn support evidence from previous observations that cold risks are highest in the oldest age group (75+ years or sometimes also starting from 65+ years) [12,19,33,34,35,36,37]. In Riga, however, the relative risk is higher in the younger age group (0–74 years, RR = 1.58 95% CI 1.12–2.22), and no statistically significant association was found in older age groups (Table 3). However, the difference between the estimates for the two age groups did not reach statistical significance in either city. When we compared the mortality rates during the winter period in Tallinn and Riga (Table 2), they were higher in the younger age group in Riga (3.2 vs. 2.6 cases per 1000 inhabitants) and higher in the older age group in Tallinn (45.7 vs. 35.1 cases per 1000 inhabitants). This indicates that there might be relatively more vulnerable people in Riga in the younger age group that die earlier because of cold temperatures. Nevertheless, stronger cold temperature associations for those younger than 65 years old have also been found in seven cities in the United States [38].

The attributable fraction of mortality that is due to cold temperatures is in line with what has previously been reported in neighboring Stockholm, Sweden, where, during the period 2000–2013, approximately 5.4% of all-cause mortality could be attributed to non-optimal cold temperatures [6]. Åström et al. [6] used year-round data, contrary to our focus on the cold season, which would imply that our estimates of the AF of cold temperatures may actually be conservative.

As was already shown in previous studies [6,7], modestly cold days affect the attributable fraction more than extremely cold days. Temperatures below the 2.5th percentile (in Tallinn −18.3 °C and in Riga −16.5 °C) were responsible for 0.6% of cold-related deaths both in Riga and in Tallinn. Although the relative risk increases with increasingly cold temperatures, most cold-related deaths occur during days with modestly cold temperatures (MMT to 2.5th percentile).

Another mortality modifying factor related to extreme temperatures is air pollution, which might have synergistic effects with ambient temperatures [39,40]. With colder weather, air pollution is often increased by extensive heating, which can modify the cold mortality results [41,42]. Chen et al. observed higher cold-related mortality risks in European urban areas experiencing high air pollution levels, with a significant modification of the effect caused by the particle number concentration (PNC) (the ultrafine range (≤100 nm) or total PNC ≤3000 nm, as a proxy for ultrafine particles) [43]. Some studies indicate that lower temperatures, in conjunction with black smoke concentrations, increase respiratory mortality [18], but, in a study of nine European cities during the cold period, no evidence for synergy with particulate matter (PM_10_), ozone (O_3_), and nitrogen dioxide (NO_2_) pollution was found [39]. As in the current study, no air pollution data was available, but this effect could be analyzed in further studies.

We were not able to run sex-specific analyses to investigate if sex modified the relationships, but earlier studies have identified that temperature-mortality effects do not differ according to sex [12,38].

One might also question how climate change may alter cold-related mortality in the future. Due to climate warming, long-term projections of temperature-related mortality in Europe predict a decrease in cold-related mortality and anticipate that this decrease will start to compensate for a rise in heat-related mortality during the second half of the 21st century [44,45]. Nevertheless, Åström et al. [6] found that, despite climate warming, the cold-related attributable fraction remained stable over time during the period 1901–2013, whereas the heat-related AF decreased in Stockholm, Sweden.

Despite the climate warming that has appeared in recent decades in Estonia [46], the cold still has a considerable effect on mortality in Estonia and Latvia, and the subject should be a constant focus of public health. During the winter period, we should pay more attention to older people because they are more vulnerable during cold days (temperatures below 4 °C). Also, we have to contribute to cardiovascular disease prevention. For prevention of cold-related health effects, the proper heating and isolation of rooms have been shown to be important [47]. Arbuthnott et al. [48] showed that comfortable indoor temperatures at night are crucial to preventing cardiovascular diseases. For preventing cold-related mortality, clothing standards for outdoor workers and home energy assistance programs for low-income households are suggested. It has been shown that policies keeping people physically active have a generally positive effect on health, and they also reduce people’s vulnerability to cold weather [47,48].

## 5. Conclusions

Cold temperatures are a serious public health concern in Riga and Tallinn. The minimum mortality temperatures during winter months in Tallinn and Riga are 4.0 °C and 4.4 °C, respectively, and, below those temperatures, mortality starts to increase. The total mortality attributable to non-optimal cold temperatures is 7.4% in Tallinn and 8.3% in Riga. It is also important to recognize that more people die during modestly cold days than extremely cold days. A significantly increased total mortality due to cold temperatures was observed in the 75+ age group, as well as due to cardiovascular mortality in Tallinn and in the below 75 age group in Riga.

## Figures and Tables

**Table 1:** Daily minimum temperatures (°C) for the winter months ^1^ over the study period for Tallinn ^2^ and Riga ^3^.

	Min	2.5th Percentile	5th Percentile	Median	Mean	SD *	95th Percentile	Max
**Tallinn**	−29.4	−18.3	−15.7	−2.6	−4.0	6.1	4.3	9.6
**Riga**	−24.4	−16.5	−14.3	−0.4	−2.1	6.1	6	8.8

^1^ Winter is defined as the period from 1 November to 31 March. ^2^ The Tallinn measurements are for the period from 1 January 1997 to 31 December 2015. ^3^ The Riga measurements are for the period from 1 January 2009 to 31 December 2015. * SD: Standard deviation.

**Table 2:** Average mortality data for the period 1997–2015 in Tallinn and 2009–2015 in Riga.

Cause of Mortality or Age Group	Average Size of Population during the Years	Number of Deaths or People in Age Group during the Whole Study Period	Annual Winter Mortality Rate (per 1000 Inhabitants)	Daily Number of Deaths				
				Mean	SD *	Median	Min	Max
**Tallinn**								
Total	402,250	36,645	4.8	12.8	4.1	12	2	29
Cardiovascular		18,941	2.5	6.6	2.7	6	0	20
Respiratory		1180	0.2	0.4	0.6	0	0	5
External		3096	0.4	1.1	1.2	1	0	8
0–74		18,320	2.6	6.4	2.7	6	0	19
75+		18,325	45.7	6.4	2.9	6	0	24
**Riga**								
Total	656,877	27,495	6.0	26.0	5.5	26	10	79
Cardiovascular		15,090	3.3	14.3	3.9	14	3	28
Respiratory		800	0.2	0.8	0.9	1	0	7
External		1673	0.4	1.6	2.1	1	0	54
0–74		13,332	3.2	12.6	3.9	12	3	65
75+		14,163	35.1	13.4	3.8	13	3	26

**Table 3:** Cumulative Relative Risks over lags of 0 to 21 days, with 95% confidence intervals.

	Tallinn		Riga	
Cause of Mortality or Age Group	RR * (95% CI)	MMT (°C)	RR (95% CI)	MMT (°C)
Total	**1.28** (1.01–1.62)	4.0	**1.41** (1.11–1.79)	4.4
Cardiovascular	**1.83** (1.31–2.55)	4.3	1.13 (0.86–1.49)	−0.9
Respiratory	2.50 (0.79–7.86)	3.3	1.37 (0.74–2.54)	−11.5
External causes	1.38 (0.63–3.02)	−1.0	1.96 (0.77–4.96)	3.2
0–74	1.08 (0.79–1.47)	2.7	**1.58** (1.12–2.22)	4.5
75+	**1.64** (1.17–2.31)	4.3	1.25 (0.90–1.73)	4.3

* RRs marked in bold indicate a statistically significant effect (*p* < 0.05).
